# The Impact of Symptoms of Depression, Anxiety, and Low Stress-Coping Capacity on the Effects of Telephone Follow-Up on Recovery Measures After Hysterectomy

**DOI:** 10.1089/whr.2023.0045

**Published:** 2024-03-27

**Authors:** Gulnara Kassymova, Gunilla Sydsjö, Ninnie Borendal Wodlin, Lena Nilsson, Preben Kjølhede

**Affiliations:** ^1^ Department of Obstetrics and Gynecology, Linköping University, Linköping, Sweden.; ^2^ Department of Biomedical and Clinical Sciences, and Linköping University, Linköping, Sweden.; ^3^ Department of Anesthesiology and Intensive Care, Linköping University, Linköping, Sweden.

**Keywords:** anxiety, depression, hysterectomy, recovery, stress coping, telephone follow-up

## Abstract

**Background:**

To investigate if symptoms of depression, anxiety, and stress-coping capacity have an impact on the effect of telephone follow-up (TFU) on trajectories of six recovery measures after hysterectomy and influence the occurrence of unplanned telephone contacts (uTCs) and unplanned visits (uVs) to health care providers.

**Material and Methods:**

A *post hoc* nonrandomized analysis of participants enrolled in a randomized, four-armed, single-blinded, controlled multicenter intervention study comprising 487 women where the women were allocated 1:1:1:1 to one of four TFU models. The Swedish Postoperative Symptom Questionnaire (SPSQ) and two health-related quality of life questionnaires, the EuroQoL-5 dimension with three levels (EQ-5 D-3 L) and the Short-Form-Health Survey (SF-36) assessed the recovery measures. The six recovery measures consisted of the EQ-5D-3L health index, the SF-36 physical component summary (PCS) and mental component summary (MCS), and the maximum and average pain intensity, and symptom sum score obtained from the SPSQ. Psychological distress was evaluated by the psychometric forms, the Hospital Anxiety and Depression Scale and the Stress Coping Inventory. The occurrence of uTC and uV within the 6 weeks of follow-up was registered.

**Results:**

Preoperative anxiety, depression, and stress-coping capacity did not modify the effects of the TFU models on the trajectories of the recovery measures, although anxiety and depression were strongly associated with all six recovery measures. uTCs, but not uVs occurred more often in the women with anxiety.

**Conclusions:**

Preoperative anxiety, depression, and stress-coping capacity did not appear to influence the effects of TFU contacts on the recovery measures after hysterectomy. Preoperative anxiety seemed to increase the occurrence of uTC. Clinical Trials Registration: ClinicalTrials.gov (NCT01526668).

## Introduction

Globally, hysterectomy is the most common major gynecological operation.^1^ Pre-existing depression and anxiety have been reported to be more common among women planned for hysterectomy than in the general female population.^2–7^ Psychological factors may influence the recovery after hysterectomy.^8–12^

Enhanced Recovery After Surgery (ERAS) programs facilitate recovery while improving outcomes. ERAS programs focus on patient education to expedite recovery, and psychological interventions can be integrated as prehabilitation.^13,14^ Today, the benefits of ERAS on the postoperative recovery are generally recognized.^15^ There is a growing awareness of the importance of psychological comorbidity in surgery and that psychological preparation may be beneficial for surgical results. However, the strength of evidence is insufficient to reach firm conclusions on the role of psychological preparation.^16–18^ The majority of publications on psychological preparations in connection with surgery deal with prehabilitation and the information on postoperative psychological interventions is scanty.

We have recently published results from a randomized multicenter study, the Post-Hysterectomy Recovery (POSTHYSTREC) study, which compared three nurse-led postoperative telephone follow-up contact (TFU) models and a control with no TFU after benign hysterectomy in an ERAS setting. No differences were seen between the TFU models in trajectories of health-related quality of life (HRQoL), postoperative symptoms, and analgesic consumption, but the TFU model, including a structured coaching, reduced the occurrence of unplanned postoperative contacts with health care providers.^19,20^

The influence of symptoms of depression and anxiety and stress-coping capacity on the effects of TFU after benign hysterectomy has not been investigated. Theoretically, TFU, especially by applying a structured coaching model, may have a positive effect on postoperative recovery in psychologically distressed women.

This study describes secondary outcomes of the POSTHYSTREC study. The aim was primarily to determine whether preoperative symptoms of anxiety and depression, and stress-coping ability have an impact on the effect of nurse-led TFU models on trajectories of recovery measures regarding HRQoL and postoperative symptoms after benign hysterectomy, and secondarily, to analyze the associations between symptoms of anxiety, depression, or stress-coping capacity and the occurrence of unplanned telephone contacts (uTCs) and unplanned visit (uVs).

## Materials and Methods

### Study setting and participants

This is *post hoc* nonrandomized analysis of participants enrolled in the randomized single-blinded controlled multicenter intervention study, the POSTHYSTREC study, which was conducted at the departments of obstetrics and gynecology in five public hospitals in the southeast health region of Sweden between October 2011 and May 2017. The POSTHYSTEC study was approved by the Regional Ethics Board of Linköping University (Dnr.2011/106-31; approval date 23 May; 2011) and conducted in accordance with the Declaration of Helsinki.^21^

A detailed study description has previously been published.^19,20^ The inclusion criteria were women aged 18–60 scheduled for abdominal or vaginal hysterectomy on benign conditions, proficiency in Swedish, and having signed the informed consent to participation after receiving written and verbal information. Exclusion criteria were genital prolapse or malignancy as surgical indication, previous or planned bilateral oophorectomy, physical or mental disability, severe psychiatric disease, and current drug or alcohol abuse.

Mode of hysterectomy (abdominal or vaginal) was selected at the surgeon's discretion in agreement with the woman, and decided before enrolment in the study. The method of anesthesia was decided by the attending anesthesiologist and complied with the current ERAS program applied in the participating clinics.

The randomization code was computer-generated^22^ with an allocation ratio of 1:1:1:1 of interventions and a stratification for center and hysterectomy type (abdominal or vaginal). The allocated intervention was written on a paper enclosed in consecutively numbered sealed envelopes. The patients were randomized before surgery in order of the numbered envelopes. The envelope was first opened upon discharge and at that time, the patient was informed about the follow-up contact model. The written and verbal information, given before inclusion, stated that TFU contact with a research nurse (RN) would be given after discharge, but the frequency or the content of the TFU was not revealed. Thus, the patients were blinded to the mode of the follow-up contacts for all other groups.

## Measures

### Demographic and clinical data

Data were collected preoperatively, and during the hospital stay. After discharge, data were gathered prospectively by the women in study-specific diaries, or obtained by the RN at the TFUs and at the 6-week end-of-study visit.

Consumption of analgesics was registered in the patient file during the hospital stay and by the patient in a diary after discharge. The daily dose of opioids was converted into an equivalent intravenous dose of morphine and that of nonopioids was converted to the World Health Organization's defined daily dose.^20,23^

### Intervention models

**FIG. 1.**
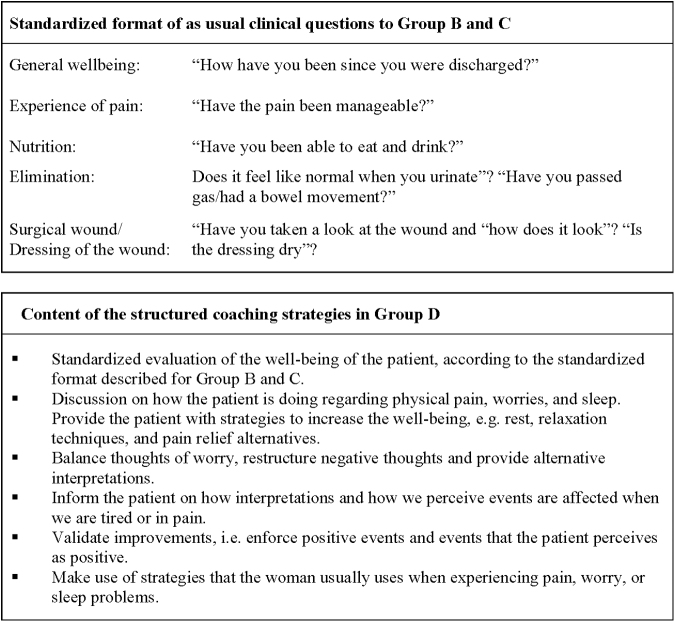
Content of standardized format of as usual clinical questions and oriented coaching telephone follow-up contact.

Patients were allocated to one of four TFU programs (Fig. 1).

- Group A - no planned follow-up contact with the health care services after discharge. The patient was requested to contact the health care services, if necessary.
- Group B - one planned, ordinary clinically structured TFU with the RN the day after discharge. Thereafter, the patient was requested to contact the health care services, if necessary.
- Group C - planned, ordinary clinically structured TFU with the RN the day after discharge and then once weekly for 6 weeks.
- Group D - planned, structured coaching TFU with the RN the day after discharge, and then once weekly for 6 weeks.

All participants were seen by the RN for collection of the questionnaires and study-specific diaries and interview at the end of the study, 6 weeks after surgery.

### Psychometric tests

The women answered two psychometric questionnaires ∼1 week before the surgery.

#### Hospital anxiety and depression scale

The hospital anxiety and depression scale (HADS) is a self-rating scale to assess psychological distress consisting of 14 items, divided into two seven-item subscales, one for anxiety (HADS-A) and one for depression (HADS-D). The scoring for each item ranges from 0 to 3, with 3 denoting the highest anxiety or depression level. The subscale summary score ranges from 0 to 21; a score ≤8 indicates no symptoms, >8 but <11 borderline symptoms, and ≥11 severe symptoms of anxiety or depression.^24^ The women were categorized according to the score for HADS-A and HADS-D scores, respectively, into three groups: *normal*, *borderline abnormal*, and *abnormal*.^25^

#### Stress-coping inventory

The stress-coping inventory (SCI) is an instrument that measures an individual's appraisal of adaptive resources for dealing with stressful situations. It describes 41 stressful situations. On a six-point Likert-type scale, the respondent rates of how often she can cope with the situation: 1- almost never; 2-rarely; 3-occasionally; 4-rather often; 5-very often; and 6-almost always. Consequently, the sum score ranges between 41 and 246. The higher the sum score, the greater is the stress-coping capacity. The women were accordingly categorized into two groups as having high and low stress-coping capacity, respectively. The cutoff level for low stress-coping capacity was set at a score ≤169.^9,26^

### Health-related quality of life

The HRQoL was assessed by two validated generic instruments, the EuroQoL-5 dimension with three levels (EQ-5D-3L) and the Short-Form-Health Survey with 36 items (SF-36).^27,28^ The health-state index of the EQ-5D-3L ranges from −0.594 to 1.000, whereas the two summary component scales scores of the SF-36 (physical component summary [PCS] and mental component summary [MCS]) range from 0 to 100.

The EQ-5D form was filled in on 14 occasions: 1 week preoperatively, then daily from the day of surgery for 8 days and thereafter once a week for 6 weeks. The SF-36 form was filled in on two occasions: 1 week preoperatively and 6 weeks after surgery.

### The Swedish postoperative symptom questionnaire

The SPSQ is a validated questionnaire that rates postoperative symptoms.^29,30^ Eight common postoperative symptoms (nausea, retching, headache, abdominal pain, tiredness, drowsiness, blurred vision, and itching) are evaluated to estimate overall discomfort.^30^ The intensity of each symptom was rated as “none” (1), “yes, a little” (2), “yes, somewhat” (3) and “yes, a lot” (4). The sum score of the eight symptoms, ranging between 8 and 32, constituted a measure of the overall discomfort; the higher the sum score the more the discomfort.

In addition, the maximum and average pain intensities are reported in the SPSQ and rated separately on a seven-point scale as: “none” (0), “very mild” (1), “mild” (2), “moderate” (3), “bad” (4), “severe” (5), and “very severe” (6).

The SPSQ questionnaire was filled in daily at the same time of the day during the first postoperative week, starting the evening after surgery, and thereafter once weekly until 6 weeks postoperatively.

### uTC and uV

uTCs and uVs, defined as any emergency or non-prearranged telephone contact or visit after discharge, for a medical reason related to the operation, with a health care provider in inpatient, outpatient, or primary care (doctors, nurses, or assistant nurses), was registered by the RN at the TFUs and at the 6-week follow-up visit.

### Outcome measures of recovery

The six outcome measures of postoperative recovery were the EQ-5D-3L health index, the SF-36 PCS and MCS, and the maximum and average pain intensity, and the symptom sum score obtained from the SPSQ.

### Statistical analyses

Data were processed in the statistical software TIBCO Statistica™, version 13.5 (TIBCO Software, Inc., Palo Alto, CA). Continuous data are presented as mean and one standard deviation, while nominal data are described as number and percentage. Univariate comparisons between groups were carried out by means of one-way analysis of variance (ANOVA) for continuous data and Pearson's chi-squared tests for nominal data.

Two-way repeated measures ANOVA models were used to analyze continuous dependent variables measured on more occasions using the nominal variable intervention and one of the ordinal categorized psychometric characteristics (HADS-A, HADS-D, or SCI) as explanatory variables. The criteria for using the repeated measures ANOVA were evaluated concerning normal distribution of continuous variables and homogeneity of variance. Since some of the continuous variables were not normally distributed, logarithmic transformations of these variables were entered into the models. *Post hoc* tests for between-group comparisons were conducted using Tukey's Honest Significant Difference tests. Logistic regression models were used to evaluate binary dependent variable measures. The statistical tests were two-sided, and the significance level was set at 5%.

#### Power analysis

The sample size estimation of the POSTHYSTREC study was based on the primary outcomes of the study (HRQoL and duration of sick leave) to obtain a significant difference between the intervention groups at a 5% level with 80% power.^19^ No *a priori* power analysis was conducted for the *post hoc* analysis concerning the relationship between psychometric characteristics and the six outcome measures. The *de facto* observed power of the psychometric characteristics on the outcome measures in the multivariable models was registered to evaluate the validity of the results.

#### Missing data

Missing data in the EQ-5D-3L on all occasions constituted 5% and in the SF-36, 0.31% and 1.04% on the two occasions of measurement. In the psychometric forms, missing data constituted 5% in the SCI and 2.9% in the HADS. For the SPSQ, the number of missing cells on all occasions made up 3.2% concerning maximum pain intensity, 3.5% for average pain intensity, and 4.1% for the items in the symptom sum score. As a repeated measures ANOVA requires data on all occasions, missing data were replaced with either the mean value or the truncated mean value of the missing cell for the specific intervention group, as appropriate.

## Results

**FIG. 2.**
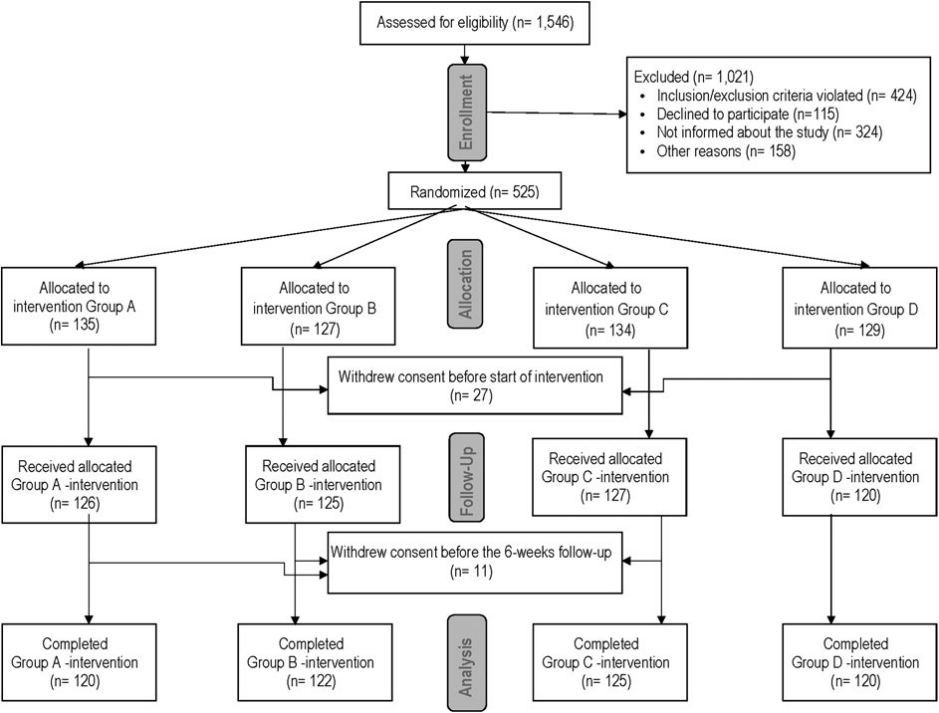
CONSORT flowchart for the POSTHYSTREC study.

The flowchart of the study population is described in Figure 2. The demographic and clinical data, and preoperative psychometric characteristics in relationship to the intervention group are presented in Table 1. The groups were balanced for all characteristics except for mental disorders, where an imbalance between the groups was observed (*p* = 0.024). Readmissions occurred in 24 (4.9%) women, equally distributed between the intervention groups.

**Table 1.** Demographic and clinical data, and preoperative psychometric data of 487 women undergoing benign hysterectomy subdivided after intervention group

	Intervention			
Characteristics	Group A (*n* = 120)	Group B (*n* = 122)	Group C (*n* = 125)	Group D (*n* = 120)
Age (years)	45.5 (5.3)	47.2 (5.6)	46.2 (5.3)	47.0 (5.8)
Categories				
< 40 years	18 (15.0)	14 (11.5)	21 (16.8)	14 (11.7)
≥ 40 - ≤ 50 years	83 (59.2)	76 (62.3)	79 (63.2)	76 (63.3)
> 50 years	19 (15.8)	32 (26.2)	25 (20.0)	30 (25.0)
BMI (kg/m^2^)	26.8 (4.8)	27.0 (4.8)	26.7 (4.6)	26.5 (4.6)
Categories				
≤ 25.0 kg/m^2^	53 (44.1)	49 (40.2)	60 (48.0)	56 (46.7)
>25.0 - < 30.0 kg/m^2^	38 (31.7)	43 (35.2)	35 (28.0)	40 (33.3)
≥30.0 kg/m^2^	29 (24.2)	30 (24.6)	30 (24.0)	24 (20.0)
Gainfully employment				
Yes	107 (89.2)	117 (95.9)	111 (88.8)	113 (94.2)
No	13 (10.8)	5 (4.1)	14 (11.2)	7 (5.8)
Comorbidity				
Mental disorders^a^	23 (19.2)	8 (6.6)	20 (16.0)	14 (11.7)
Chronic pain disorder	28 (23.3)	30 (24.6)	29 (23.2)	31 (25.8)
Taking medicine regularly preoperatively				
Analgesics				
Non opioid analgesics	14 (11.7)	20 (16.4)	22 (17.6)	11 (9.2)
Opioid analgesics	4 (3.3)	3 (2.5)	6 (4.8)	6 (5.0)
Hypnotics	8 (6.7)	2 (1.6)	7 (5.6)	7 (5.8)
Previously undergone laparotomy	39 (32.8)	37 (30.5)	46 (37.4)	39 (32.8)
Hysterectomy indication				
Myoma uteri	58 (48.3)	65 (53.3)	47 (37.6)	53 (44.2)
Bleeding disorder	32 (26.7)	23 (18.8)	35 (28.0)	35 (29.2)
Myoma and bleeding	10 (8.3)	14 (11.5)	21 (16.8)	13 (10.8)
Cervical dysplasia	14 (11.7)	12 (9.8)	14 (11.2)	9 (7.5)
Pain	5 (4.2)	8 (6.6)	8 (6.4)	9 (7.5)
Others	1 (0.8)	0 (0.0)	0 (0.0)	1 (0.8)
Mode of hysterectomy				
Abdominal	97 (80.8)	98 (80.3)	98 (77.4)	90 (75.0)
Vaginal	23 (19.2)	24 (19.7)	27 (21.6)	30 (25.0)
Mode of anesthesia				
GA	55 (45.8)	37 (30.3)	50 (40.0)	41 (34.2)
ITA + IT morphine	34 (28.4)	52 (42.6)	45 (36.0)	49 (40.8)
GA + IT morphine	31 (25.8)	33 (27.1)	30 (24.0)	30 (25.0)
Intraoperative complications^b^	7 (5.8)	8 (6.6)	3 (2.4)	3 (2.5)
Hospital stay (days)	1.8 (1.1)	1.8 (1.3)	1.6 (1.0)	1.6 (1.2)
Day of discharge^c^				
Day 1	47 (39.2)	53 (43.4)	50 (40.0)	64 (53.3)
Day 2	60 (50.0)	55 (45.1)	62 (49.6)	48 (40.0)
Day 3	8 (6.7)	10 (8.2)	7 (5.6)	7 (5.8)
Day 4 or later	5 (4.2)	4 (3.3)	6 (4.8)	1 (0.8)
Postoperative complications (Clavien-Dindo)^d^				
I	17 (14.2)	15 (12.3)	16 (12.8)	9 (7.5)
II	17 (14.2)	19 (15.6)	24 (19.2)	15 (12.5)
III	3 (2.5)	6 (4.9)	4 (3.2)	4 (3.3)
Readmissions within 6 weeks postoperatively	3 (2.5)	7 (5.7)	6 (4.8)	8 (6.7)
Analgesic consumption				
Opioids (equivalent iv. morphine [mg])				
Day 2	2.9 (5.8)	3.3 (7.4)	3.8 (6.3)	2.8 (5.6)
Day 3	2.1 (4.7)	1.8 (4.6)	2.2 (4.6)	2.2 (4.8)
Day 4	1.4 (3.8)	1.8 (6.5)	1.6 (4.3)	1.5 (3.7)
Day 5	0.8 (2.7)	1.3 (5.3)	1.0 (3.0)	0.9 (3.6)
Day 6	0.7 (2.6)	0.9 (4.9)	0.7 (2.6)	0.2 (1.1)
Day 7	0.4 (1.7)	0.9 (4.6)	0.7 (2.6)	0.4 (2.5)
Nonopioids (DDD)				
Day 2	2.1 (1.0)	2.2 (1.1)	2.1 (1.1)	2.1 (1.2)
Day 3	1.9 (1.1)	1.9 (1.1)	2.0 (1.1)	2.0 (1.1)
Day 4	1.9 (1.1)	1.7 (1.1)	1.8 (1.1)	1.9 (1.1)
Day 5	1.7 (1.1)	1.6 (1.2)	1.6 (1.1)	1.7 (1.1)
Day 6	1.5 (1.1)	1.4 (1.1)	1.5 (1.1)	1.4 (1.2)
Day 7	1.4 (1.1)	1.4 (1.1)	1.4 (1.1)	1.2 (1.1)
Day 8	1.2 (1.1)	1.2 (1.0)	1.2 (1.1)	1.1 (1.1)
Day 9	1.1 (1.0)	1.1 (1.0)	1.0 (1.0)	1.0 (1.1)
Day 10	0.9 (0.9)	1.0 (1.0)	0.9 (0.9)	0.9 (1.0)
Day 11	0.8 (0.9)	0.9 (0.9)	0.8 (0.9)	0.8 (1.0)
Day 12	0.7 (0.8)	0.8 (0.9)	0.8 (0.9)	0.7 (0.9)
Day 13	0.5 (0.8)	0.7 (0.9)	0.6 (0.9)	0.6 (0.8)
Day 14	0.5 (0.7)	0.6 (0.8)	0.6 (0.8)	0.5 (0.8)
Day 15	0.4 (0.7)	0.4 (0.7)	0.5 (0.8)	0.4 (0.8)
Psychometric characteristics preoperatively				
EQ-5D-3L health index				
mean score	0.79 (0.22)	0.79 (0.21)	0.79 (0.22)	0.80 (0.18)
SF-36				
PCS	46.9 (10.6)	47.1 (9.9)	48.1 (9.1)	48.0 (8.5)
MCS	47.2 (10.7)	46.4 (10.3)	47.0(11.1)	48.0 (10.1)
HADS–A				
mean score	5.3 (4.1)	5.0 (4.1)	4.9 (4.1)	4.6 (3.9)
Categories				
Normal (score 0–7)	81 (67.5)	93 (76.2)	92 (73.6)	92 (76.7)
Borderline (score 8–10)	24 (20.0)	15/12.3)	17 (13.6)	17 (14.2)
Abnormal (score 11–21)	15 (12.5)	14 (11.5)	16 (12.8)	11 (9.2)
HADS–D				
mean score	2.7 (2.8)	3.1 (2.4)	2.6 (3.1)	2.6 (2.8)
Categories				
Normal (score 0–7)	107 (89.2)	104 (85.3)	117 (93.6)	110 (91.7)
Borderline (score 8–10)	13 (10.8)	12 (9.8)	5 (4.0)	6 (5.0)
Abnormal (score 11–21)	0 (0.0)	6 (4.9)	3 (2.4)	4 (3.3)
SCI				
mean score	187.0 (25.4)	183.6 (25.2)	185.0 (26.7)	187.5 (24.9)
Categories				
Low stress coping (score ≤169)	32 (26.7)	33 (27.0)	27 (21.6)	30 (25.0)
High stress coping (score >169)	88 (73.3)	89 (73.0)	98 (78.4)	90 (75.0)

Figures denote mean and (standard deviation) or number of women and (percent).

^a^ Mental disorders comprise minor mental disorders such as anxiety, panic, depressive and mood disorders, and phobias.

^b^ Intraoperative complications include intraoperative bleeding exceeding 1000 mL, large vessel or organ damage.

^c^ Day of discharge: Day of surgery = Day 0.

^d^ Clavien-Dindo classification of postoperative complications, contracted form.

ASA, American Society of Anesthesiologists; DDD, defined daily dose; EQ-5D-3L, EuroQol Group – five dimensions and three levels form; GA, general anesthesia; HADS-D, Hospital Anxiety and Depression Scale–Depression; HADS-A, Hospital Anxiety and Depression Scale–Anxiety; IT, intrathecal; ITA, intrathecal anesthesia; MCS, mental component summary; PCS, physical component summary; SCI, Stress Coping Inventory; SF-36, 36-item Short Form Health Survey.

### Impact of psychometric characteristics on interventions

Because there was no statistically significant effect of the various interventions that is, the TFU programs, on the outcome measures, the association of psychometric characteristics with the various outcome measures was analyzed. The analyses, using intervention and psychometric characteristic as explanatory variables, showed no differences between the intervention groups in any of the six outcome measures, the EQ-5D-3L health index, the SF-36 PCS and MCS, the maximum and average pain intensity, and SPSQ symptom sum score (Table 2). All outcome measures, except the SF-36's PCS for all psychometric characteristics and MCS for HADS-A, improved statistically significantly over time (effect over time).

**Table 2.** Association between the intervention groups and the six outcome measures of recovery in relationship to psychometric measures

	Outcome measure	Crude^a^/Adjusted^b^	Between intervention groups	Within groups		Between intervention and psychometric measure
			Main effect	Effect over time^c^	Interaction effect^c^	Interaction effect^c^
			*p*-value	*p*-value	*p*-value	*p*-value
HADS-A	EQ-5D-3L health index	Crude	0.78	<0.0001	0.38	0.47
		Adjusted	0.68	<0.0001	0.28	0.71
	SF-36 PCS	Crude	0.26	<0.0001	0.73	0.68
		Adjusted	0.13	0.52	0.52	0.47
	SF-36 MCS	Crude	0.33	<0.001	0.44	0.15
		Adjusted	0.32	0.07	0.49	0.22
	Maximum pain intensity	Crude	0.64	<0.0001	0.57	0.76
		Adjusted	0.68	<0.0001	0.32	0.79
	Average pain intensity	Crude	0.67	<0.0001	0.52	0.80
		Adjusted	0.81	<0.0001	0.60	0.95
	Symptom sum score	Crude	0.88	<0.0001	0.57	0.53
		Adjusted	0.77	<0.0001	0.70	0.43
HADS D	EQ-5D-3L health index	Crude	0.79	<0.0001	0.52	0.21
		Adjusted	0.99	<0.0001	0.79	0.55
	SF-36 PCS	Crude	0.09	<0.0001	0.65	0.11
		Adjusted	0.24	0.79	0.59	0.17
	SF-36 MCS	Crude	0.35	<0.0001	0.80	0.82
		Adjusted	0.54	<0.01	0.75	0.87
	Maximum pain intensity	Crude	0.10	<0.0001	0.97	0.19
		Adjusted	0.36	<0.0001	0.98	0.63
	Average pain intensity	Crude	0.25	<0.0001	0.57	0.60
		Adjusted	0.58	<0.0001	0.98	0.92
	Symptom sum score	Crude	0.86	<0.0001	0.15	0.07
		Adjusted	0.14	<0.0001	0.29	0.17
SCI	EQ-5D-3L health index	Crude	0.76	<0.0001	0.14	0.35
		Adjusted	1.00	<0.0001	0.85	0.38
	SF-36 PCS	Crude	0.35	<0.0001	0.62	0.88
		Adjusted	0.23	0.33	0.47	0.80
	SF-36 MCS	Crude	0.23	<0.01	0.78	0.18
		Adjusted	0.64	0.047	0.72	0.27
	Maximum pain intensity	Crude	0.92	<0.0001	0.70	0.73
		Adjusted	0.96	<0.0001	0.73	0.71
	Average pain intensity	Crude	0.91	<0.0001	0.36	0.88
		Adjusted	0.78	<0.0001	0.61	0.92
	Symptom sum score	Crude	0.93	<0.0001	0.16	0.77
		Adjusted	0.84	<0.0001	0.45	0.80

^a^ Two-way repeated measures ANOVA. Analysis includes two explanatory variables (intervention and HADS-A, HADS-D or SCI, respectively).

^b^ Two-way repeated measures ANOVA. Besides the two explanatory variables, the models were adjusted for mode of surgery, day of discharge (category), consumption of opioids day 2–7, and nonopioids day 2–15.

^c^ Sphericity violated. Greenhouse-Geisser correction used for the variables effect over time and interaction effects.

No interaction effect was observed between the interventions over time in any of the outcome measures, indicating that the outcome measures changed equally over time in the intervention groups. Neither was any interaction effects found between the psychometric measures and the interventions in any of the outcome measures. This indicates that neither of the interventions seemed to be favored by a particular psychometric characteristic. The observed power of the psychometric characteristics in the repeated measures ANOVA models varied for the six outcome variables between 0.72 and 1.00 (median 0.92, interquartile range 0.83–1.00) concerning HADS-A, between 0.66 and 1.00 (median 0.71, interquartile range 0.66–0.99) concerning HADS-D, and between 0.19 and 1.00 (median 0.53, interquartile range 0.31–0.97) concerning SCI.

### Association between psychometric characteristics and outcome measures

In the adjusted models (adjustment for TFU models (intervention), mode of surgery, day of discharge, and opioid and nonopioid consumption), HADS-A and HADS-D were significantly associated (main effect of psychometric characteristics) with all six outcome measures, whereas SCI was only associated with the symptom sum score and the SF-36's MCS (Supplementary Table S1). No interaction effects were seen between the psychometric characteristics and the outcome measures, except for all three psychometric measures concerning SF-36 MCS and for HADS-D concerning EQ-5D-3L health index, and for SCI concerning the symptom sum score. The significant associations between the psychometric characteristics and the outcome measures reported in Supplementary Table S1 are graphically illustrated in Supplementary Figures 1–3. The *post hoc* tests revealed that the differences in outcome measures between the HADS-A and the HADS-D groups were mainly attributed to significant differences between the categories *normal* and *abnorma*l (*p*-values between 0.02 and <0.0001).

The category *normal* reported higher HRQoL measures, lower pain intensity, and symptom sum scores than the category *abnormal*. Corresponding findings were observed between the HADS-A groups *normal* and *borderline abnormal* concerning EQ-5D-3L health index (*p* = 0.03), and symptom sum score (*p* < 0.0001), and between *borderline abnormal* and *abnormal* for SF-36 MCS (*p* < 0.0001). Even the HADS-D groups demonstrated differences between *normal* and *borderline abnormal* in SF-36 PCS (*p* = 0.02) and SF-36 MCS (*p* < 0.0001) and between *borderline abnormal* and *abnormal* in SF-36 MCS (*p* = 0.046). For SCI, the high stress-coping capacity group had higher HRQoL scores in the HRQoL measures EQ-5D-3L (*p* < 0.01) and SF-36 MCS (*p* < 0.0001) and lower symptom sum score (*p* < 0.0001) than the low stress-coping capacity group.

### Impact of psychometric characteristics on uTC and uV

In total, 224 (46.0%) women had uTCs and 218 (44.8%) had uVs. The analyses revealed significant associations between uTC and HADS-A and HADS-D scores but not for SCI score (Table 3), whereas uV only was associated with HADS-D score (Table 3). A higher score of HADS-A or HADS-D was positively correlated to a higher rate of uTCs. In terms of categories of HADS-A and –D, the associations were mainly attributed to *abnormal* anxiety (aOR 2.28, 95% CI: 1.22–4.27) and *borderline abnormal* depression (aOR 2.97, 95% CI: 1.28–6.90).

**Table 3.** Association between psychometric tests (HADS-A, HADS-D, and SCI) and unplanned telephone contacts (A) and unplanned visits (B), respectively, after benign hysterectomy

A	Unplanned telephone contacts (number of women)		Logistic regression			
			Univariate	Multivariable^a^		
	Yes (*n* = 224)	No (*n* = 263)	(OR and 95% CI)	Model includes HADS-A (aOR and 95% CI)	Model includes HADS-D (aOR and 95% CI)	Model includes SCI (aOR and 95% CI)
Intervention						
Group A	57 (25.4)	63 (24.0)	Reference	Reference	Reference	Reference
Group B	65 (29.0)	57 (21.8)	1.26 (0.76–2.09)	1.34 (0.77–2.32)	1.26 (0.72–2.18)	1.30 (0.75–2.24)
Group C	63 (28.1)	63 (24.0)	1.09 (0.66–1.80)	1.08 (0.63–1.87)	1.05 (0.61–1.82)	1.04 (0.61–1.70)
Group D	40 (17.9)	80 (30.4)	0.55 (0.33–0.93)	0.63 (0.36–1.11)	0.61 (0.34–1.06)	0.61 (0.35–1.07)
HADS-A						
Score	5.6 (4.3)	4.4 (3.8)	1.08 (1.03–1.13)	1.08 (1.02–1.13)	—	—
HADS-D						
Score	3.2 (3.1)	2.4 (3.0)	1.09 (1.03–1.16)	—	1.09 (1.02–1.17)	—
SCI						
Score	184.8 (25.1)	186.6 (26.0)	1.00 (0.99–1.00)	—	—	1.00 (0.99–1.00)
Age						
>50 years	39 (17.4)	67 (25.5)	Reference	Reference	Reference	Reference
41–50 years	151 (67.4)	163 (62.0)	1.59 (1.01–2.50)	1.64 (0.99–2.70)	1.63 (0.99–2.68)	1.67 (1.02–2.74)
≤40 years	34 (15.2)	33 (12.5)	1.77 (0.95–3.29)	1.58 (0.78–3.21)	1.57 (0.78–3.18)	1.70 (0.84–3.41)
BMI						
<25 kg/m^2^	95 (42.4)	123 (46.8)	Reference	Reference	Reference	Reference
25–29.9 kg/m^2^	74 (33.0)	82 (31.1)	1.17 (0.77–1.77)	1.17 (0.74–1.85)	1.18 (0.75–1.86)	1.20 (0.77–1.89)
≥30 kg/m^2^	55 (24.6)	58 (22.1)	1.23 (0.78–1.94)	1.10 (0.66–1.83)	1.08 (0.65–1.80)	1.14 (0.69–1.89)
Hysterectomy						
Abdominal	183 (81.7)	200 (76.0)	Reference	Reference	Reference	Reference
Vaginal	41 (18.3)	63 (24.0)	0.71 (0.41–1.11)	0.73 (0.44–1.20)	0.72 (0.44–1.18)	0.75 (0.46–1.23)
Day of discharge						
Day 1	91 (40.6)	123 (46.8)	Reference	Reference	Reference	Reference
Day 2	106 (47.3)	119 (45.2)	1.20 (0.83–1.75)	0.84 (0.55–1.29)	0.86 (0.56–1.32)	0.88 (0.57–1.34)
Day 3	17 (7.6)	15 (5.7)	1.53 (0.73–3.23)	0.82 (0.34–1.96)	0.86 (0.36–2.03)	0.85 (0.36–2.02)
Day 4 or later	10 (4.5)	6 (2.3)	2.25 (0.79–6.42)	0.81 (0.25–2.59)	0.81 (0.25–2.60)	0.83 (0.26–2.64)
Postoperative complications^b^						
0	116 (51.8)	222 (84.4)	Reference	Reference	Reference	Reference
I	41 (18.3)	16 (6.1)	4.90 (2.64–9.11)	4.70 (2.48–8.90)	4.71 (2.49–8.91)	4.76 (2.52–8.99)
II	55 (24.5)	20 (7.6)	5.26 (3.01–9.20)	5.19 (2.87–9.37)	5.13 (2.85–9.26)	5.21 (2.90–9.36)
III	12 (5.4)	5 (1.9)	4.59 (1.58–13.35)	5.19 (1.64–16.44)	5.35 (1.69–16.90)	5.07 (1.59–16.13)

B	Unplanned visits (number of women)		Logistic regression			
			Univariate	Multivariable^a^		
	*Yes (*n* = 218)*	*No (*n* = 269)*	(OR and 95% CI)	Model includes HADS-A (aOR and 95% CI)	Model includes HADS-D (aOR and 95% CI)	Model includes SCI (aOR and 95% CI)
Intervention						
Group A	57 (26.2)	63 (23.4)	Reference	Reference	Reference	Reference
Group B	54 (24.8)	68 (25.3)	0.88 (0.53–1.46)	0.82 (0.45–1.51)	0.78 (0.43–1.44)	0.81 (0.44–1.49)
Group C	62 (28.4)	63 (23.4)	1.09 (0.66–1.80)	1.00 (0.55–1.83)	0.98 (0.54–1.78)	0.98 (0.54–1.77)
Group D	45 (20.6)	75 (27.9)	0.66 (0.40–1.11)	0.80 (0.44–1.48)	0.77 (0.42–1.42)	0.79 (0.43–1.45)
HADS-A						
Score	5.5 (4.3)	4.5 (3.8)	1.07 (1.02–1.11)	1.06 (1.00–1.12)	—	—
HADS-D						
Score	3.1 (3.3)	2.5 (2.8)	1.07 (1.01–1.14)	—	1.06 (0.99–1.14)	—
SCI						
Score	184.9 (25.3	186.4 (25.8)	1.00 (0.99–1.00)	—	—	1.00 (0.99–1.01)
Age						
>50 years	38 (17.4)	68 (25.3)	Reference	Reference	Reference	Reference
41–50 years	143 (65.6)	171 (63.6)	1.50 (0.95–2.36)	1.58 (0.91–2.71)	1.57 (0.91–2.70)	1.60 (0.93–2.75)
≤40 years	37 (17.0)	30 (11.1)	2.21 (1.18–4.12)	2.00 (0.93–4.34)	1.99 (0.92–4.29)	2.08 (0.97–4.47)
BMI						
<25 kg/m^2^	89 (40.8)	47 (17.5	Reference	Reference	Reference	Reference
25–29.9 kg/m^2^	63 (28.9)	93 (34.6)	0.98 (0.65–1.49)	1.06 (0.64–1.76)	1.08 (0.65–1.78)	1.09 (0.66–1.80)
≥30 kg/m^2^	66 (30.3)	129 (47.9)	2.04 (1.28–3.23)	2.34 (1.35–4.05)	2.35 (1.36–4.07)	2.42 (1.40–4.18)
Hysterectomy						
Abdominal	178 (81.7)	205 (76.2)	Reference	Reference	Reference	Reference
Vaginal	40 (18.3)	64 (23.8)	0.72 (0.46–1.21)	0.80 (0.46–1.37)	0.79 (0.46–1.36)	0.81 (0.47–1.39
Day of discharge						
Day 1	76 (34.9)	138 (51.3)	Reference	Reference	Reference	Reference
Day 2	111 (50.9)	114 (42.4)	1.77 (1.21–2.59)	1.26 (0.80–2.00)	1.29 (0.81–2.04)	1.30 (0.82–2.06)
Day 3	19 (8.7)	13 (4.8)	2.65 (1.24–5.67)	1.29 (0.49–3.38)	1.33 (0.52–3.43)	1.34 (0.52–3.44)
Day 4 or later	12 (5.5)	4 (1.5)	5.45 (1.70–17.48)	1.55 (0.41–5.92)	1.65 (0.41–5.91)	1.56 (0.41–6.00)
Postoperative complications^b^						
0	95 (43.6)	243 (90.3)	Reference	Reference	Reference	Reference
I	42 (19.3)	15 (5.6)	7.16 (3.79–13.52)	6.97 (3.60–13.48)	7.00 (3.6–13.54)	7.03 (3.64–13.56)
II	67 (30.7)	8 (3.0)	21.42 (9.91–46.29)	19.95 (9.01–44.18)	19.68 (8.89–43.55)	19.86 (8.9843.91)
III	14 (6.4)	3 (1.1)	11.94 (3.35–42.48)	13.38 (3.46–51.79)	13.79 (3.56–53.39)	13.29 (3.42–51.60)

Figures denote number and (percent) or mean and (standard deviation).

^a^ Adjusted for intervention, age, BMI, hysterectomy method, day of discharge, postoperative complications, and HADS-A, HADS-D, or SCI, respectively.

^b^ Clavien-Dindo classification of postoperative complications, contracted form.

aOR, adjusted odds ratio; BMI, body mass index; CI, confidence interval; OR, odds ratio.

Concerning uV, a higher score of HADS-A was associated with an increased occurrence of uV, although this could not be attributed to a particular category of anxiety. Postoperative complications were unanimously very strong independent risk factors of both uTC and uV that seemed to even up the effect of the other risk factors disclosed in the univariate analyses. In addition, a BMI ≥30 kg/m^2^, classified as obesity, constituted an independent risk factor for uV, but not for uTC.

## Discussion

The main findings of the study were that the psychometric characteristics HADS-A, HADS-D, and SCI did not modify the effects of the TFU interventions on the trajectories of the recovery measures maximum and average pain, the sum score of eight common postoperative symptoms, and generic HRQoL measures. Although the psychometric characteristics did not modify the effect of the interventions, all outcomes were associated with the HADS-A and HADS-D, whereas only the symptom sum score and the MCS of SF-36 were associated with the SCI.

The effect of the interventions on the occurrence of uTCs was evened up by the HADS-A, HADS-D, and SCI. HADS-A and HADS-D, but not SCI, were associated with uTCs, whereas only HADS-A was associated with uV. Adjusted for the psychometric measures, uTC and uV were both strongly associated with the occurrence of postoperative complications. Obesity did not affect the occurrence of uTCs but was a strong predictor of uVs.

We believe this to be the first study that has evaluated the impact of TFU on the trajectories of postoperative recovery measurements after benign hysterectomy in relationship to symptoms of depression, anxiety, and stress coping capacity.

Surgery is a stressful situation that can trigger feelings of anxiety and depression. These feelings can make it more difficult for the individual to cope with the stress and it can affect recovery. Higher presurgical depression and lower self-efficacy to manage illness have been associated with poorer trajectories of recovery after different surgical procedures.^31–33^ Low stress-coping capacity negatively affected postoperative recovery after hysterectomy conducted in an ERAS setting.^9^ Preoperative anxiety, depression, and low self-efficacy are consistently associated with worse physiological surgical outcomes and postoperative quality of life.^17^ Psychological preparation may be beneficial for postoperative pain and behavioral recovery,^16^ and has been transferred into trimodal prehabilitation programs, including nutritional, physical, and psychological preparation before surgery.^34^

In connection with major abdominal surgery, trimodal prehabilitation improved physical functioning, preoperative HADS score, and reduced anxiety.^35,36^ However, it cannot be ruled out that the lack of effect of the TFU on recovery measures of the groups with symptoms of anxiety, depression, and low stress-coping capacity in this study may be due to the use of an ERAS program and the incorporated preoperative psychological preparation that has been practiced for many years in the participating hospitals.

Coaching strategies of self-care are often based on principles of cognitive behavioral therapy (CBT). Coaching is intended to help the individual to manage negative thoughts and experiences and reframe beliefs, values, perceptions, rules, and assumptions that have become obstacles to achieving the individual's goals, for instance for a smooth recovery after surgery. CBT is a recognized method for treatment of stress, anxiety, and depression symptoms. It is therefore conceivable that a structured coaching based on CBT principles postoperatively may help individuals to achieve the goal of a smooth recovery.

Nurse coaching has mostly been studied in connection with chronic illness and the outcome variables were often physiological and biochemical measures in addition to quality of life and psychometric assessments.^37,38^ Only a few studies that have dealt with postoperative care could be compared to the present study. A postoperative telephone coaching concept after outpatient breast cancer surgery improved physical functioning and reduced emotional distress 3 weeks postsurgery.^39^ Nurse coaching by telephone after ambulatory arthroscopy significantly reduced postoperative symptom distress and improved HRQoL 1 week after surgery.^40^ However, the follow-up times in both these studies were shorter than in the present study.

The use of psychological interventions by psychologists postoperatively improved emotional stress and immune response in women with breast cancer.^41^ Thus, the lack of coaches who were professionally educated in psychology in the present study could be an explanation for why no effects were seen on the outcome measures in the TFU group with structured coaching in women with psychological distress. However, a survey study among certified nurse coaches and advanced practice nurses has stated that nurse practitioners may be strategically situated to provide coaching and have the knowledge and skills needed to intervene with medically complex, at-risk populations.^42^

uTC and uV can be seen as a request for advice or help for a patient who experiences concerns about a deviation in the postoperative recovery, for example, occurrence of unexpected symptoms, increased symptom intensity, or unexpected slow recovery in relationship to the anticipated postoperative course. The readmission rate in the present study was low, and did not differ between the groups. Thus, the TFUs *per se* did not appear to influence or prevent the risk of readmission. A positive consequence of uTCs and uVs may be that they may prevent medically unnecessary readmissions.

The relatively high number of women who had uTCs and uVs in the present study in relationship to the low readmission rate may support this assumption. Although the number of women with uTCs was significantly lower in the group that received coaching TFUs, the effect of coaching TFU on uTC seemed to be outweighed by the effect of the psychometric property, especially symptoms of anxiety or depression, which became independent risk factors for uTC.

Neither the frequency of complications nor the grade of complications according to the Clavien-Dindo classification differed between the intervention groups A-D.^19^ Preoperative anxiety is a predictor of postoperative pain.^43–45^ We have previously shown that pain and other distressing postoperative symptoms were the main causes for uTCs and uVs preceded by a uTC.^20^ Thus, our finding that uTC and uV was more frequent in women with higher anxiety levels seemed to support this association. Contrarily, the association between preoperative depression and postoperative pain is ambiguous.^46^ This may explain why we found an association between uTC and the *borderline abnormal*, but not with the *abnormal* HADS-D group.

A major strength is the randomized design of the original study and the large number of participants. Another strength is the use of commonly used and validated instruments for psychometric, HRQoL, and postoperative symptom assessments. The study has limitations. The content of the TFUs has not been strictly validated. The study constitutes a *post hoc* analysis of nonrandomized subgroups of participants. Besides the risk of selection bias in such design, this implies a risk of reduced power compared with the power estimated for the original randomized study and thus a risk of misleading interpretation of findings. Consequently, the results must be interpreted with caution. However, the analyses revealed a fairly high observed power, in particular for anxiety and depression symptoms, indicating that the conclusions of these outcomes seem reasonable.

The most common method of follow-up after benign hysterectomy in Sweden is the use of the patient's self-reported questionnaire from the Swedish National Quality Register of Gynecological Surgery 8 weeks after the surgery. All clinics offer TFU or in-person follow-up visits on the woman's request, and a few clinics practice TFU or follow-up visits routinely. The intervention group D received an experimental treatment using a structured coaching model based on elements derived from CBT. We thus believe that the follow-up methods have a solid clinical and theoretical foundation. Another potential limitation might be survey fatigue. The rates of missing data were in general low, and we found no significant tendency of increased missing data over time.

A possible reason for not achieving an effect on the recovery measures of the TFUs, in particular, when adding a structured coaching intervention, with women with symptoms of depression, anxiety, and lower stress-coping capacity may be that all groups having TFUs were contacted and/or coached by the same study nurses. This might have influenced the content of the TFU in groups C and D, where a confusion or mixing of coaching strategy and the usual clinical strategy for the RNs cannot be ruled out. However, to prevent such unintended effect, GS had several education sessions with the RNs, emphasizing the importance of separating the structured oriented coaching TFU from the standard follow-up regimens.

Understanding the impact of mental health, particularly depression and anxiety, on the postoperative recovery may help with developing strategies for preoperative psychological preparation and tailoring postoperative follow-up to expedite recovery and increase HRQoL in women after hysterectomy. Well-designed randomized studies are needed to evaluate such strategies and interventions. TFUs with or without coaching are resource-demanding and the cost-effectiveness of follow-up interventions after surgery needs to be analyzed before implementation in clinical routine.^38,39^

## Conclusion

Symptoms of anxiety and depression and stress-coping capacity preoperatively did not seem to have impact on the effect of nurse-led TFU with regard to postoperative symptoms and recovery of HRQoL after benign hysterectomy in an ERAS setting. Preoperative symptoms of anxiety seemed to increase the occurrence of uTCs, but not the occurrence of uVs.

## Supplementary Material

Supplemental data

Supplemental data

Supplemental data

Supplemental data

## Data Availability

Data are available on reasonable request and in accordance with Swedish legislation from the corresponding author.

## Abbreviations Used

ANOVA: analysis of variance
ASA: American Society of Anesthesiologists
CBT: cognitive behavioral therapy
DDD: defined daily dose
EQ-5D-3L: EuroQol Group – five dimensions and three levels form
ERAS: enhanced recovery after surgery
GA: general anesthesia
HADS: hospital anxiety and depression scale
HADS-A: Hospital Anxiety and Depression Scale–Anxiety
HADS-D: Hospital Anxiety and Depression Scale–Depression
HRQoL: health-related quality of life
MCS: mental component summary
PCS: physical component summary
POSTHYSTREC: posthysterectomy recovery
RN: research nurse
SCI: stress-coping inventory
SF-36: 36-item Short Form Health Survey
SPSQ: Swedish postoperative symptom questionnaire
TFU: telephone follow-up
uTC: unplanned telephone contact
uV: unplanned visit
