# Supplementary material for: The Impact of Symptoms of Depression, Anxiety, and Low Stress-Coping Capacity on the Effects of Telephone Follow-Up on Recovery Measures After Hysterectomy
Source: Womens Health Rep (New Rochelle). 2024 Mar 27;5(1):304–18. doi: 10.1089/whr.2023.0045 (PMC10979684; doi:10.1089/whr.2023.0045)
Supplement: Supplemental data [file Supp_TableS1.docx]

Supplemental Table 1. Association between the psychometric groups of HADS-A, HADS-D and SCI, respectively, and the six recovery outcome measures.

|  |  | | Between psychometric groups | Within groups | |
| --- | --- | --- | --- | --- | --- |
|  |  | | Main effect | Effect over time ^c^ | Interaction effect ^c^ |
|  | Outcome measure | Crude ^a^ / Adjusted ^b^ | p-value | p-value | p-value |
| HADS-A | EQ-5D-3L health index | Crude | <0.001 | <0.0001 | 0.19 |
|  |  | Adjusted | <0.0001 | <0.0001 | 0.09 |
|  | SF-36 PCS | Crude | 0.02 | <0.0001 | 0.05 |
|  |  | Adjusted | 0.01 | 0.55 | 0.06 |
|  | SF-36 MCS | Crude | <0.0001 | <0.001 | <0.001 |
|  |  | Adjusted | <0.0001 | 0.03 | <0.001 |
|  | Maximum pain intensity | Crude | 0.01 | <0.0001 | 0.66 |
|  |  | Adjusted | <0.01 | <0.0001 | 0.79 |
|  | Average pain intensity | Crude | <0.01 | <0.0001 | 0.14 |
|  |  | Adjusted | <0.01 | <0.0001 | 0.21 |
|  | Symptom sum score | Crude | <0.0001 | <0.0001 | 0.88 |
|  |  | Adjusted | <0.0001 | <0.0001 | 0.98 |
| HADS D | EQ-5D-3L health index | Crude | 0.04 | <0.0001 | <0.01 |
|  |  | Adjusted | 0.02 | <0.0001 | <0.01 |
|  | SF-36 PCS | Crude | <0.01 | <0.0001 | 0.02 |
|  |  | Adjusted | <0.01 | 0.79 | 0.08 |
|  | SF-36 MCS | Crude | <0.0001 | <0.0001 | <0.0001 |
|  |  | Adjusted | <0.0001 | <0.01 | <0.0001 |
|  | Maximum pain intensity | Crude | 0.01 | <0.0001 | 0.82 |
|  |  | Adjusted | 0.03 | <0.0001 | 0.92 |
|  | Average pain intensity | Crude | <0.01 | <0.0001 | 0.74 |
|  |  | Adjusted | 0.03 | <0.0001 | 0.70 |
|  | Symptom sum score | Crude | <0.0001 | <0.0001 | 0.70 |
|  |  | Adjusted | <0.0001 | <0.0001 | 0.91 |
| SCI | EQ-5D-3L health index | Crude | <0.01 | <0.0001 | 0.71 |
|  |  | Adjusted | 0.02 | <0.0001 | 0.79 |
|  | SF-36 PCS | Crude | 0.02 | <0.0001 | 0.53 |
|  |  | Adjusted | 0.08 | 0.36 | 0.58 |
|  | SF-36 MCS | Crude | <0.0001 | <0.01 | <0.001 |
|  |  | Adjusted | <0.0001 | 0.047 | <0.0001 |
|  | Maximum pain intensity | Crude | 0.14 | <0.0001 | 0.49 |
|  |  | Adjusted | 0.29 | <0.0001 | 0.35 |
|  | Average pain intensity | Crude | 0.07 | <0.0001 | 0.76 |
|  |  | Adjusted | 0.15 | <0.0001 | 0.85 |
|  | Symptom sum score | Crude | <0.0001 | <0.0001 | 0.01 |
|  |  | Adjusted | <0.001 | <0.0001 | 0.01 |

EQ-5D-3L, EuroQol Group – five dimensions and three levels form; HADS-A, Hospital Anxiety and Depression Scale – Anxiety; HADS-D, Hospital Anxiety and Depression Scale – Depression; SCI, Stress Coping Inventory; SF-36, Short-Form-36; MCS, mental component summary; PCS, physical component summary.

^a^ Two-way repeated measures ANOVA. Analysis includes two explanatory variables (intervention and HADS-A, HADS-D or SCI, respectively).

^b^ Two-way repeated measures ANOVA. Besides the two explanatory variables, the models were adjusted for mode of surgery, day of discharge (category), consumption of opioids day 2-7, and non-opioids day 2-15.

^c^ Sphericity violated. Greenhouse-Geisser correction for the variables effect over time and interaction effect.
