# Supplementary material for: The Impact of Symptoms of Depression, Anxiety, and Low Stress-Coping Capacity on the Effects of Telephone Follow-Up on Recovery Measures After Hysterectomy
Source: Womens Health Rep (New Rochelle). 2024 Mar 27;5(1):304–18. doi: 10.1089/whr.2023.0045 (PMC10979684; doi:10.1089/whr.2023.0045)
Supplement: Supplemental data [file Supp_FigS1.pdf]

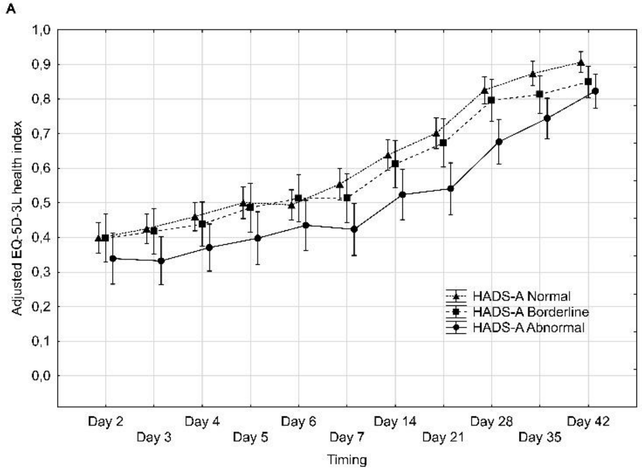

Post hoc tests:		
	Normal vs Borderline:	p= 0.03
	Borderline vs Abnormal:	p= NS
	Normal vs Abnormal:	p<0.0001

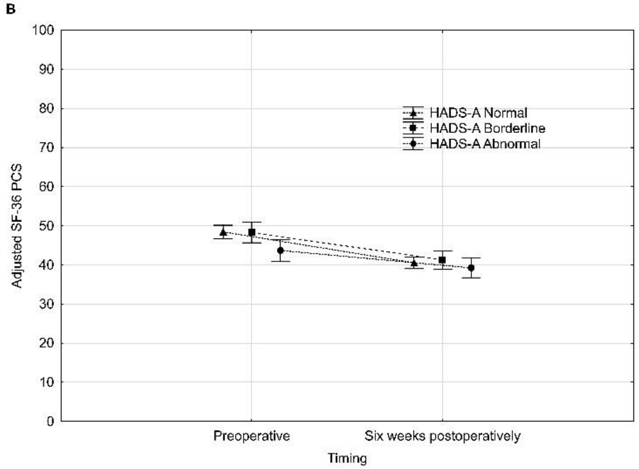

Post hoc tests:		
	Normal vs Borderline:	p= NS
	Borderline vs Abnormal:	p= NS
	Normal vs Abnormal:	p<0.01

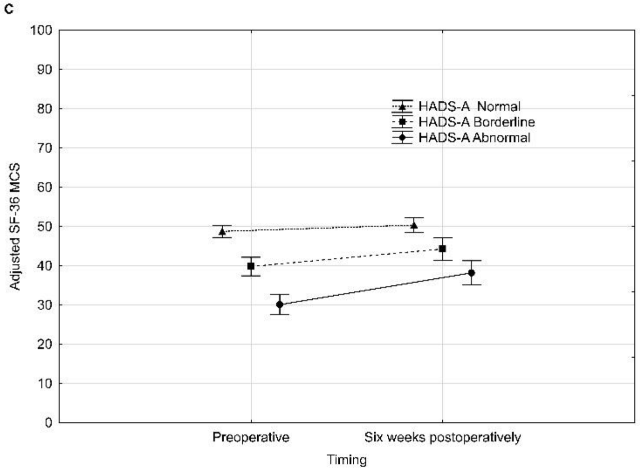

Post hoc tests:		
	Normal vs Borderline:	p<0.0001
	Borderline vs Abnormal:	p<0.0001
	Normal vs Abnormal:	p<0.0001

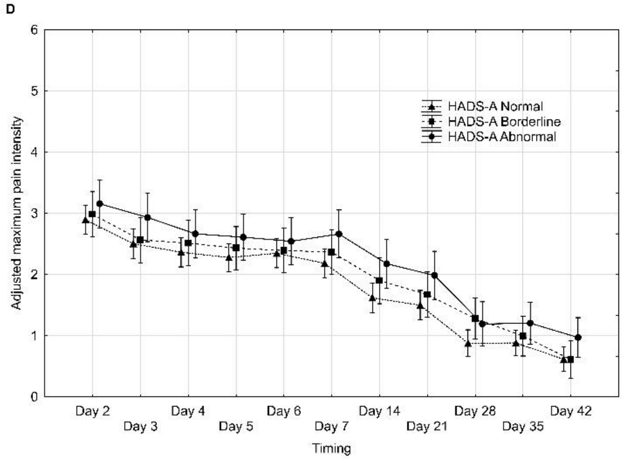

Post hoc tests:		
	Normal vs Borderline:	p= NS
	Borderline vs Abnormal:	p= NS
	Normal vs Abnormal:	p=0.01

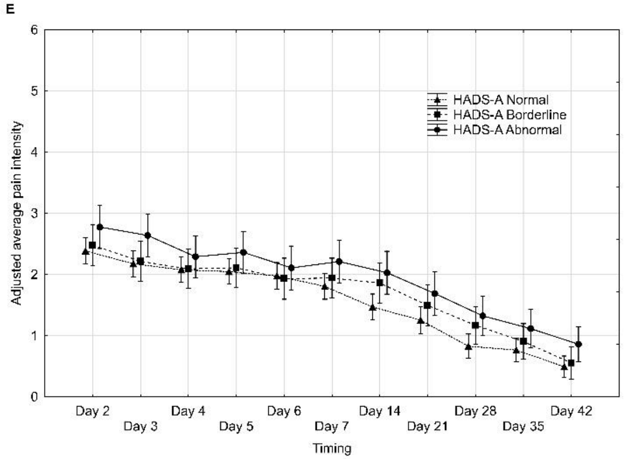

Post hoc tests:		
	Normal vs Borderline:	p= NS
	Borderline vs Abnormal:	p= NS
	Normal vs Abnormal:	p<0.01

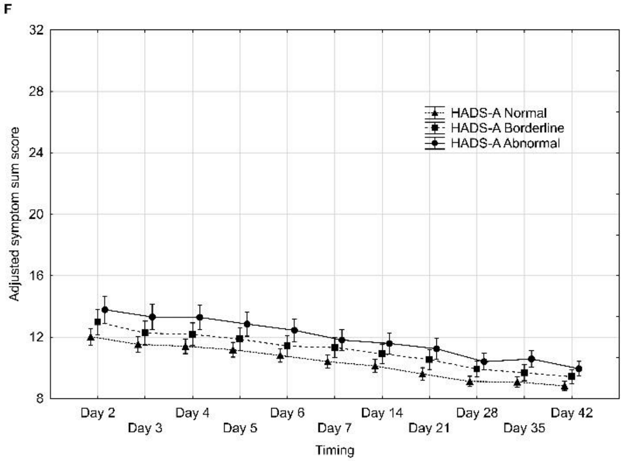

Post hoc tests:		
	Normal vs Borderline:	p<0.0001
	Borderline vs Abnormal:	p= NS
	Normal vs Abnormal:	p<0.0001

Supplemental Figure 1. Graphic presentation of the trajectory of measurements of the dependent variables in relation to category of HADS-A. Plots indicate mean, and bars indicate 95% confidence interval. The p-values of the post hoc tests are reported below each figure. NS = not significant.
