# Supplementary material for: The Impact of Symptoms of Depression, Anxiety, and Low Stress-Coping Capacity on the Effects of Telephone Follow-Up on Recovery Measures After Hysterectomy
Source: Womens Health Rep (New Rochelle). 2024 Mar 27;5(1):304–18. doi: 10.1089/whr.2023.0045 (PMC10979684; doi:10.1089/whr.2023.0045)
Supplement: Supplemental data [file Supp_FigS3.pdf]

M

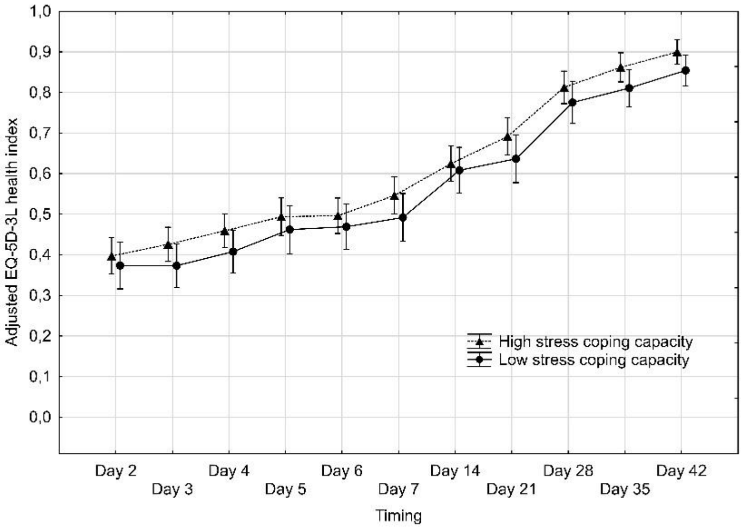

Post hoc test: High vs Low stress coping p<0.01

O

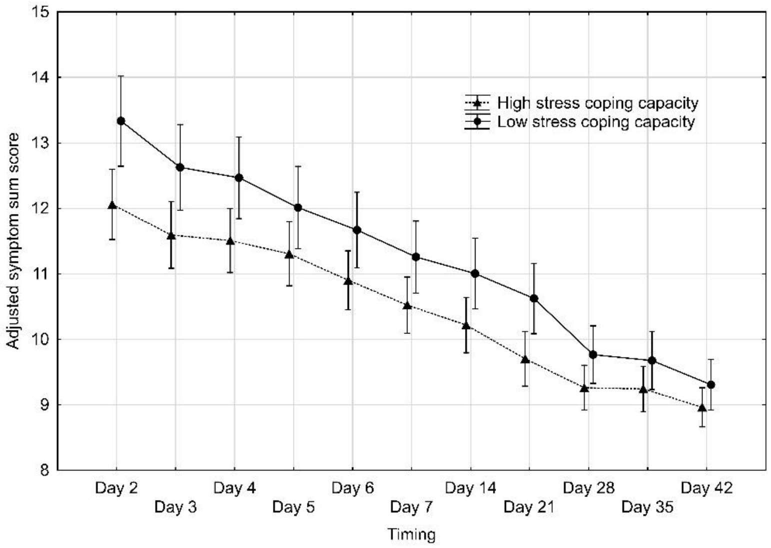

Post hoc test: High vs Low stress coping p<0.0001

N

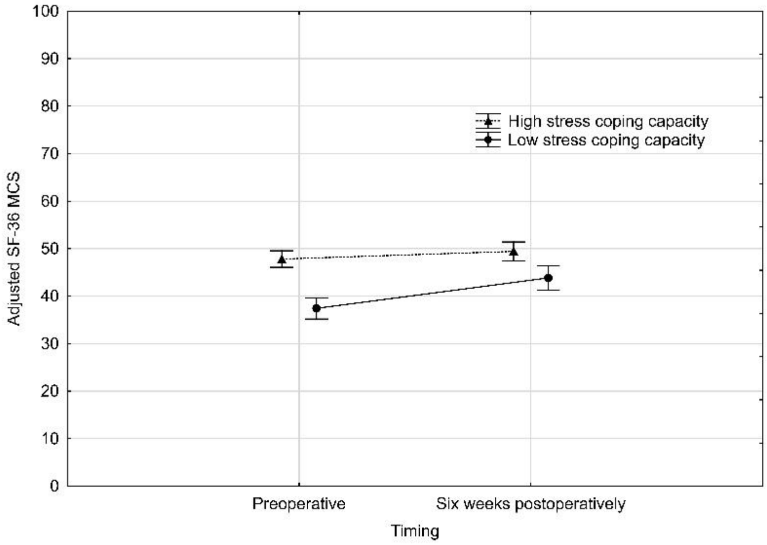

Post hoc test: High vs Low stress coping p<0.01:

Supplemental Figure 3. Graphic presentation of the trajectory of measurements of the dependent variables in relation to category of SCI. Only the variables that were found to be statistical significant in the adjusted models (Supplemental Table 1) are presented. Plots indicate mean, and bars indicate 95% confidence interval. The p-values of the post hoc tests are reported below each figure.
