# Supplementary material for: The Impact of Symptoms of Depression, Anxiety, and Low Stress-Coping Capacity on the Effects of Telephone Follow-Up on Recovery Measures After Hysterectomy
Source: Womens Health Rep (New Rochelle). 2024 Mar 27;5(1):304–18. doi: 10.1089/whr.2023.0045 (PMC10979684; doi:10.1089/whr.2023.0045)
Supplement: Supplemental data [file Supp_FigS2.pdf]

G

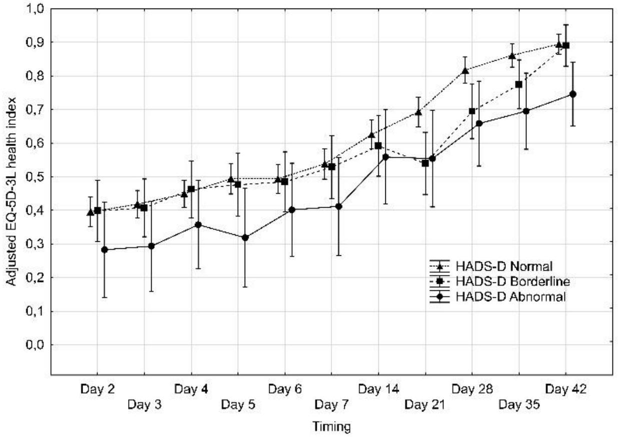

Post hoc tests:
Normal vs Borderline: p= NS
Borderline vs Abnormal: p= NS
Normal vs Abnormal: p=0.01

H

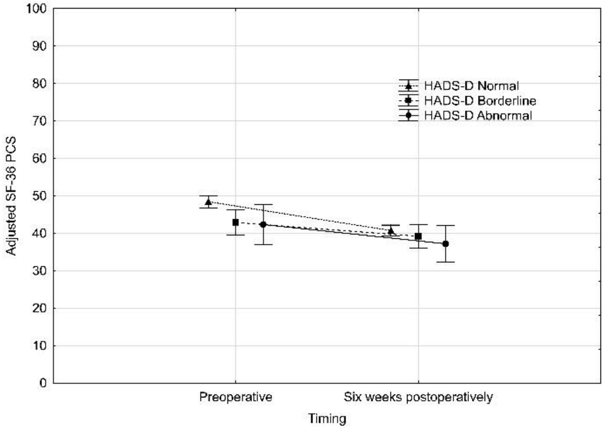

Post hoc tests:
Normal vs Borderline: p= 0.02
Borderline vs Abnormal: p= NS
Normal vs Abnormal: p<=0.02

I

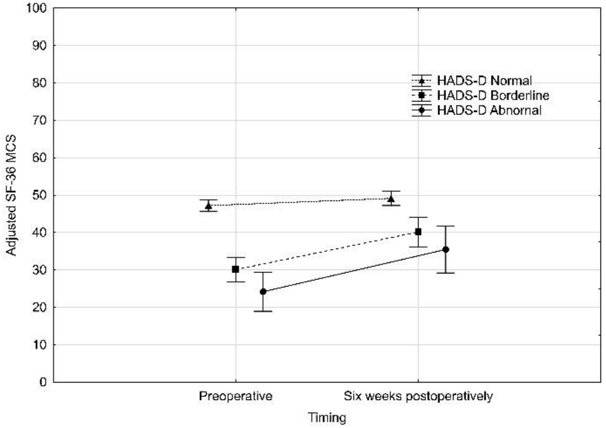

Post hoc tests:
Normal vs Borderline: p<0.0001
Borderline vs Abnormal: p=0.047
Normal vs Abnormal: p<0.0001

J

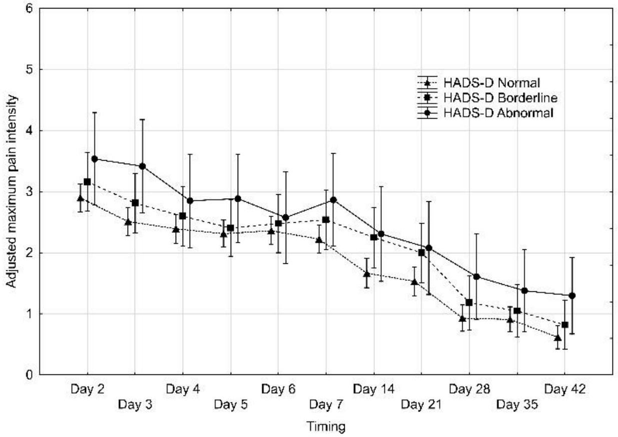

Post hoc tests:
Normal vs Borderline: p= NS
Borderline vs Abnormal: p= NS
Normal vs Abnormal: p<0.01

K

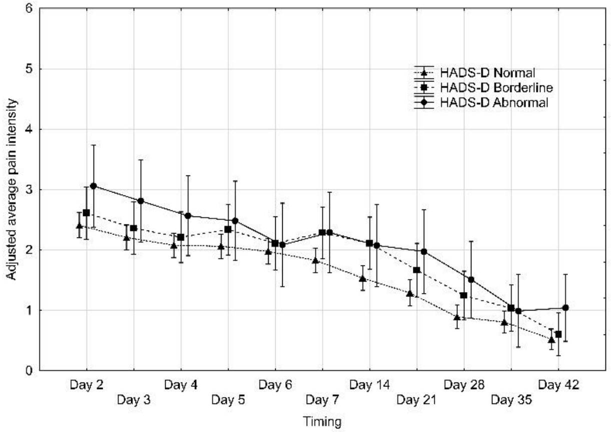

Post hoc tests:
Normal vs Borderline: p= NS
Borderline vs Abnormal: p= NS
Normal vs Abnormal: p<0.01

L

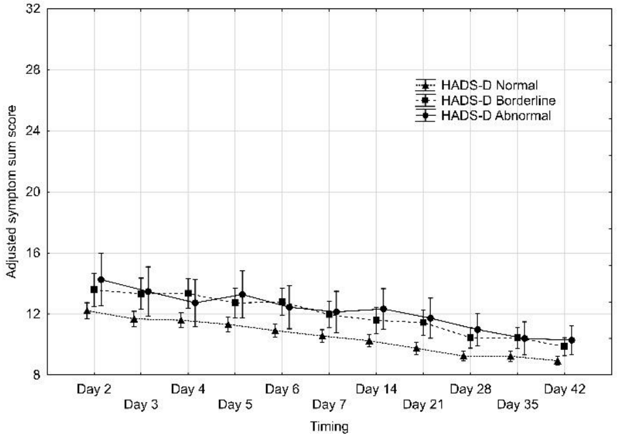

Post hoc tests:
Normal vs Borderline: p<0.0001
Borderline vs Abnormal: p= NS
Normal vs Abnormal: p<0.001

Supplemental Figure 2. Graphic presentation of the trajectory of measurements of the dependent variables in relation to category of HADS-D. Plots indicate mean, and bars indicate 95% confidence interval. The p-values of the post hoc tests are reported below each figure. NS = not significant.
